# Using a consensus technique for improving the methodology of clinical trials assessing treatments for Cutaneous Leishmaniasis

**DOI:** 10.1186/1745-6215-16-S2-P59

**Published:** 2015-11-16

**Authors:** Astrid Erber, Liliana Lopez-Carvajal, Byron Arana, Trudie Lang, Piero Olliaro

**Affiliations:** 1 University of Oxford, Oxford, UK; 2 Universidad de Antioquia, Medellin, Colombia; 3 Drugs for Neglected Diseases Initiative (DNDi), Geneva, Switzerland; 4 World Health Organization Special Programme for Research and Training in Tropical Diseases (WHO - TDR), Geneva, Switzerland

## 

Cutaneous Leishmaniasis (CL) is a tropical parasitic disease in humans, transmitted by sandflies. CL causes lesions on exposed parts of the body, potentially leaving life-long scars. The disease manifestations vary significantly, depending on parasite species. An estimated 0.7 - 1.3 million new cases occur worldwide annually.

Recommendations on the treatment of CL currently have a weak evidence base: not only are treatment options limited, but also have systematic reviews pointed to a lack of methodological standardization in the conduct and analysis of clinical trials of CL interventions.

Standardized clinical trial methodologies would provide clinical investigators with guidance for the design, conduct, analysis and report of future clinical trials of treatments for CL. This includes the definition of measurable, reproducible and clinically meaningful outcomes. In order for both current and newer treatments to be adequately assessed, standardised methodologies are needed which can be applied generally, while at the same time allowing the flexibility required to cover the diverse disease manifestations.

A guidance document on the methodology of clinical trials assessing CL interventions was published and provides the basis for further refinement. For this project, an iterative online Delphi consensus methodology is used. It involves stakeholders in order to agree on a set of core outcomes and eligibility criteria for CL treatment trials. The targeted stakeholder groups are researchers and health care providers (physicians and nurses) working with CL, and treating CL patients. Additional input is being sought from patients via interviews, to reflect their perspectives.

CL is used as a case-study to assess whether the key stakeholders, most of them in disease endemic countries, can be identified and encouraged to collaborate using an innovative, participatory approach. The project is in progress; preliminary results will be presented.

